# L-Asparaginase Conjugates from the Hyperthermophilic Archaea *Thermococcus sibiricus* with Improved Biocatalytic Properties

**DOI:** 10.3390/ijms25084174

**Published:** 2024-04-10

**Authors:** Natalia V. Dobryakova, Maria V. Dumina, Alexander A. Zhgun, Marina V. Pokrovskaya, Svetlana S. Aleksandrova, Dmitry D. Zhdanov, Elena V. Kudryashova

**Affiliations:** 1 Group of Fungal Genetic Engineering, Federal Research Center “Fundamentals of Biotechnology of the Russian Academy of Sciences”, 117312 Moscow, Russia; natdobryak@gmail.com (N.V.D.); duminamaria@gmail.com (M.V.D.); zhdanovdd@gmail.com (D.D.Z.); 2 Chemical Faculty, Lomonosov Moscow State University, Leninskie Gory St. 1, 119991 Moscow, Russia; 3 Laboratory of Medical Biotechnology, Institute of Biomedical Chemistry, 119121 Moscow, Russia

**Keywords:** L-asparaginase, polymer conjugates, *Thermococcus sibiricus*, CD spectrometry

## Abstract

This study investigated the effect of polycationic and uncharged polymers (and oligomers) on the catalytic parameters and thermostability of L-asparaginase from *Thermococcus sibiricus* (TsA). This enzyme has potential applications in the food industry to decrease the formation of carcinogenic acrylamide during the processing of carbohydrate-containing products. Conjugation with the polyamines polyethylenimine and spermine (PEI and Spm) or polyethylene glycol (PEG) did not significantly affect the secondary structure of the enzyme. PEG contributes to the stabilization of the dimeric form of TsA, as shown by HPLC. Furthermore, neither polyamines nor PEG significantly affected the binding of the L-Asn substrate to TsA. The conjugates showed greater maximum activity at pH 7.5 and 85 °C, 10–50% more than for native TsA. The pH optima for both TsA-PEI and TsA-Spm conjugates were shifted to lower pH ranges from pH 10 (for the native enzyme) to pH 8.0. Additionally, the TsA-Spm conjugate exhibited the highest activity at pH 6.5–9.0 among all the samples. Furthermore, the temperature optimum for activity at pH 7.5 shifted from 90–95 °C to 80–85 °C for the conjugates. The thermal inactivation mechanism of TsA-PEG appeared to change, and no aggregation was observed in contrast to that of the native enzyme. This was visually confirmed and supported by the analysis of the CD spectra, which remained almost unchanged after heating the conjugate solution. These results suggest that TsA-PEG may be a more stable form of TsA, making it a potentially more suitable option for industrial use.

## 1. Introduction

**Figure 1:**
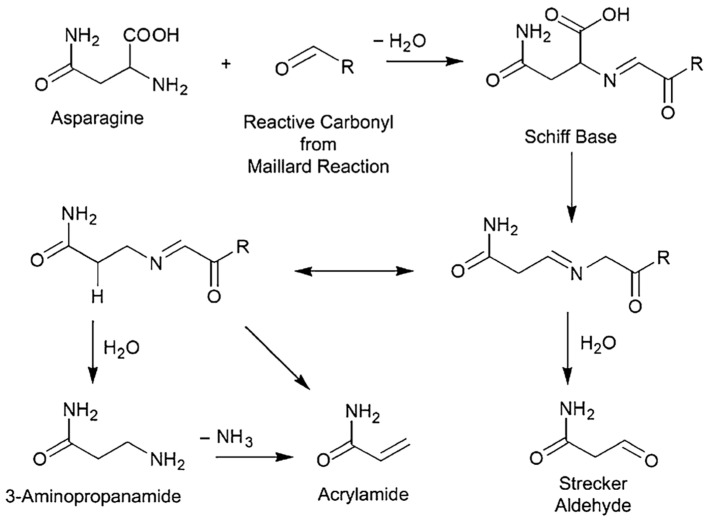
This figure shows the formation of acrylamide during the reaction of reducing sugars with L-asparagine [6].

The study of L-asparaginases (L-ASNases; E.C. 3.5.1.1.) from thermophilic sources is an important task that aims to improve the properties of enzymes for application in the food and pharmaceutical industries. L-ASNase is commonly used as a processing agent in the food industry to reduce acrylamide levels in commercial fried foods while maintaining their color, flavor, and texture [1,2]. L-ASNase catalyzes the hydrolysis of L-asparagine (L-Asn), which helps to prevent its reaction with sugars and the formation of acrylamide. Acrylamide is a potentially carcinogenic substance formed when reducing sugars react with L-Asn (Maillard reaction, Figure 1) at temperatures above 100–120 °C during frying, baking, and roasting [3,4,5]. To reduce the concentration of L-Asn in products, foods containing high amounts of sugars can be treated with L-ASNase. This enzymatic treatment can result in less acrylamide formation during food preparation.

However, the stability of existing commercial L-ASNase is limited, which may affect their application in certain fields. *Aspergillus fungi* are commonly used in the baking industry, but their enzyme activity may decrease at temperatures above 100–120 °C [7]. Hence, one of the main issues is the search for new sources of L-ASNases with improved thermal stability. It is worth considering hyperthermophilic organisms, such as the archaea *Thermococcus sibiricus*, as potential sources for this purpose [8]. This L-ASNase from archaea has optimum activity at 90 °C and can be used for industrial applications.

One approach to regulating the catalytic properties and thermostability of enzymes is through the formation of conjugates with polymers [9,10]. Immobilization prevents denaturation and aggregation of the protein at elevated temperatures and preserves its activity. This was successfully achieved upon covalent modification of L-ASNases from different sources [11,12,13,14,15]. Previous studies have demonstrated the efficacy of modifying the L-ASNases of *Erwinia carotovora* (EwA) and *Rhodospirillum rubrum* (RrA) with polycations such as graft polymers based on chitosan and polyethylenimine (chitosan-PEG, chitosan-glycol, PEI-PEG, chitosan-PEI) [16,17,18]. Polyamines were observed to be effective at modifying RrA. The conjugation of RrA with PEI derivatives, including PEI-PEG, resulted in higher activity at pH 7.5, improved thermostability, and increased resistance to trypsinolysis in comparison to the unmodified enzyme. The aim of this study was to investigate the possibility of regulating the catalytic properties of *Thermococcus sibiricus* L-ASNase by producing conjugates of the enzyme with polymers of different compositions. This study compared the effects of PEI, PEG, and spermine on the structural properties, activity, and thermostability of the enzyme.

## 2. Results

### 2.1. Characteristics of TsA Conjugates

The aim of this study was to investigate how the physicochemical properties and functional activity of TsA are affected by the formation of conjugates with PEI and Spm polyamines in comparison to uncharged PEG. Additionally, the effects of polycations on L-ASNases from various sources have been investigated [18]. For example, in the case of the EwA enzyme, the conjugate with PEG-chitosan was found to be the most effective, exhibiting enhanced activity and thermostability. In contrast, for RrA, PEI-PEG and Spm were identified as the most effective compounds [16,17]. Therefore, it is crucial to compare the impacts of charged and uncharged polymers on this enzyme. Table 1 presents a brief overview of the polymers used in this study.

**Figure 2:**
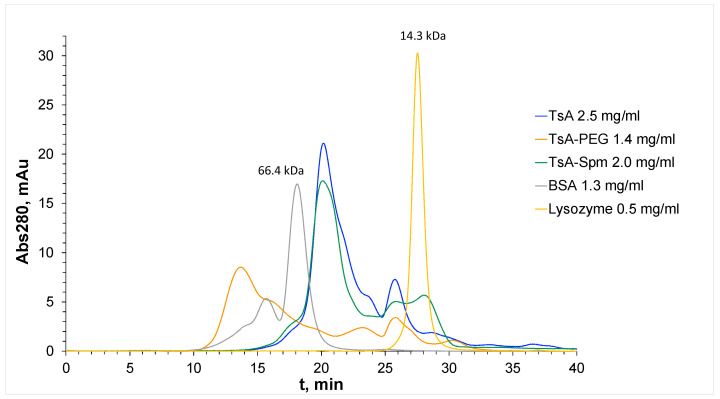
Chromatograms of TsA, TsA-Spm, and TsA-PEG conjugate solutions. Eluent—50 mM Tris buffer containing 0.2 M NaCl, pH 7.5.

To monitor the modification of the conjugates, HPLC gel filtration chromatography was performed on the obtained samples. Figure 2 displays chromatograms of the native TsA and the modified TsA variants, while Table 2 shows the retention times of the fractions. The reference samples used were solutions of BSA with MM = 66.4 kDa and lysozyme with MM = 14.3 kDa. The peak with the highest intensity (catalytic activity is detectable) for the initial TsA was located at 21 min, which corresponds to the monomeric form of the protein with MM = 37.5 kDa [8].

The TsA-PEG conjugate displayed the main peak at 14 min, indicating a pronounced increase in MM, as a result of the covalent attachment of PEG chains. Electrophoresis (SDS-PAGE) of the TsA-PEG conjugate (Figure S1) also revealed the formation of the TsA-PEG conjugate (a wide band at approximately 75–94 kDa) and the absence of the band at 37–40 kDa (TsA monomeric form) observed for the native enzyme. For the TsA-Spm conjugate, only small changes in the chromatogram were detected after the modifications. This can be explained by the low molecular weight of spermine, which makes only a small impact on the molecular weight of the conjugate.

**Figure 3:**
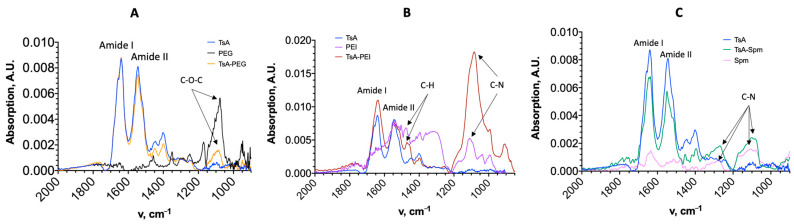
IR spectra of the native enzyme (TsA) and conjugates and free polymers: (**A**) TsA-PEG, (**B**) TsA-PEI, (**C**) TsA-Spm.

The TsA conjugates were characterized using IR and CD spectroscopy. Figure 3 shows the IR spectra of the native enzyme, the conjugates, and the polymers.

The spectra of all conjugates revealed peaks corresponding to amide I (1600–1700 cm^−1^) and amide II (1500–1600 cm^−1^), which are characteristic of peptide bonds in proteins. As shown in Figure 3A, both the TsA-PEG conjugate and PEG itself exhibit a peak at 1075 cm^−1^, which corresponds to the C-O-C bond vibrations of polyethylene glycol. Figure 3B displays a characteristic peak at 1085–1110 cm^−1^ (C-N vibrations) for both free PEI and the TsA-PEG conjugate, with TsA-PEG having the most intense peak, confirming the modification. In Figure 3C, peaks at 1250 and 1060–1120 cm^−1^ (C-N vibrations) are present in the TsA-Spm conjugate, consistent with the presence of Spm. The number of polymer chains per enzyme molecule in the conjugates was estimated by analyzing the IR spectra. For TsA-PEG, the intensity of the peak at 1089 cm^−1^ yields approximately 5–6 PEG chains. The degree of modification can be determined using the titration method with TNBS, which has been shown to yield comparable results. For TsA-PEI and TsA-Spm, the intensity of the peak at 1100 cm^−1^ yields 7–8 chains and 15 chains correspondingly.

**Figure 4:**
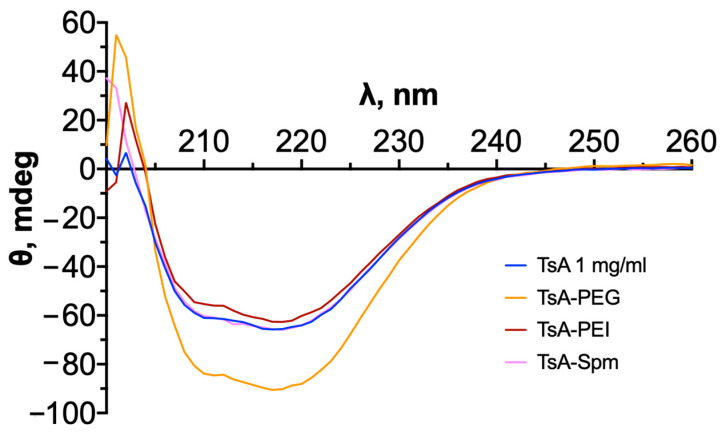
CD spectra of the native enzyme and TsA-PEG, TsA-PEI, and TsA-Spm conjugates. C(TsA) = 1 mg/mL (0.027 mM), 0.01 M PBS, 37 °C, pH 7.5.

The resulting conjugates were subsequently compared to the native enzyme for determination of their secondary structure content via CD spectroscopy. Visual and quantitative analysis of the CD spectra (Figure 4) indicated that the conjugates retained their secondary structure after modification (Table 3). 

**Figure 5:**
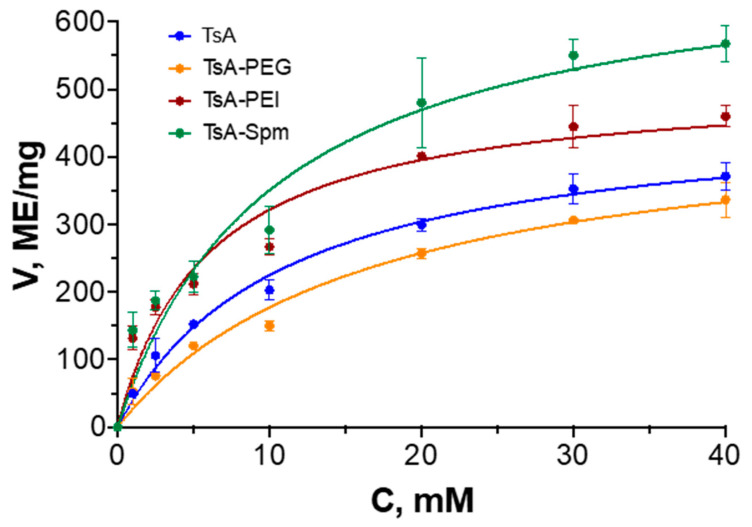
Dependences of hydrolysis reaction rate on L-Asn concentration. pH 7.5, 85 °C. The concentration of the enzyme in the final solution was 0.007 mg/mL (0.19 μM).

To evaluate the effects of L-ASNase modification on the catalytic activity and substrate binding efficiency, the maximum reaction rate (Vmax) and Michaelis constant (K_M_) were calculated. The kinetic curves for TsA and the conjugates are shown in Figure 5, while Table 4 shows the kinetic parameters of the enzymes at 85 °C.

The data presented suggest that the modification of TsA by polymers has no significant effect on the binding of the substrate to the enzyme (K_M_ values). However, the maximum rate of the hydrolysis reaction changes significantly under the indicated conditions. Notably, while Vmax did not change for the TsA-PEG conjugate, it is much higher for TsA-PEI and TsA-Spm than for the native enzyme (470 IU/mg). The highest value was observed for the TsA-Spm conjugate (710 IU/mg).

### 2.2. pH Dependence of TsA Conjugate Activity Compared to That of Native L-ASNase

**Figure 6:**
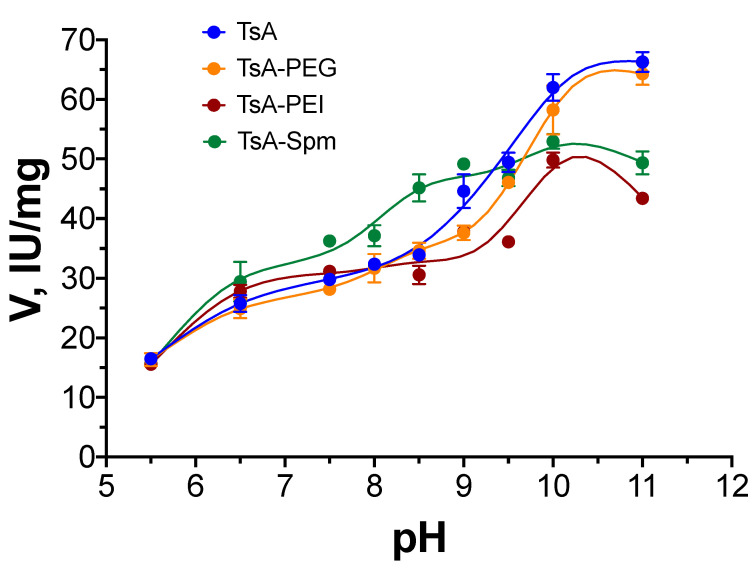
The pH dependence of TsA and the TsA-PEG, TsA-PEI, and TsA-Spm conjugates. Conditions: 37 °C, 20 mM L-Asn, and 5 mM citrate–phosphate–borate buffer.

The data presented suggest that TsA conjugates may exhibit increased activity under conditions similar to those used in the industrial application of L-ASNases for this enzyme. Notably, conjugation with polycations resulted in a shift in the optimum pH for activity in the direction of lower pH, as observed, for example, for EwA conjugates [16]. Hence, it is essential to compare the pH dependence profiles of TsA and its conjugates at lower temperatures (37 °C). This comparison may help in understanding how activity could be affected under conditions close to those observed in biotechnological processes. This is important for assessing the potential of these enzymes in biomedical or bioanalytical applications at pH 7.5 and 37 °C. The results of determining the pH dependence profiles of TsA and its conjugates are presented in Figure 6.

The activity of the native enzyme and the conjugate with the uncharged polymer TsA-PEG showed similar pH profiles, with optimum pH activity observed at pH 10–10.5. However, there was a difference in activity profiles for conjugates with PEI and Spm polycations. TsA-PEI showed an activity optimum at pH 10. The pH dependence profile for TsA-Spm was flatter than that for the native enzyme. The highest activity achieved for the conjugate with Spm was 40 IU/mg, which was achieved at neutral pH 7.5–8.5 and 37 °C, while the native enzyme had lower activity at this pH (~30 IU/mg). So, the conjugate with spermine exhibited the highest activity in the biotechnologically relevant (physiological) pH range of 7.0–9.0. This characteristic is particularly important for practical purposes, such as the blanching process of food products, or biomedical analysis which is typically carried out at a pH 7.0–7.5.

### 2.3. Temperature Dependence of the Activity

**Figure 7:**
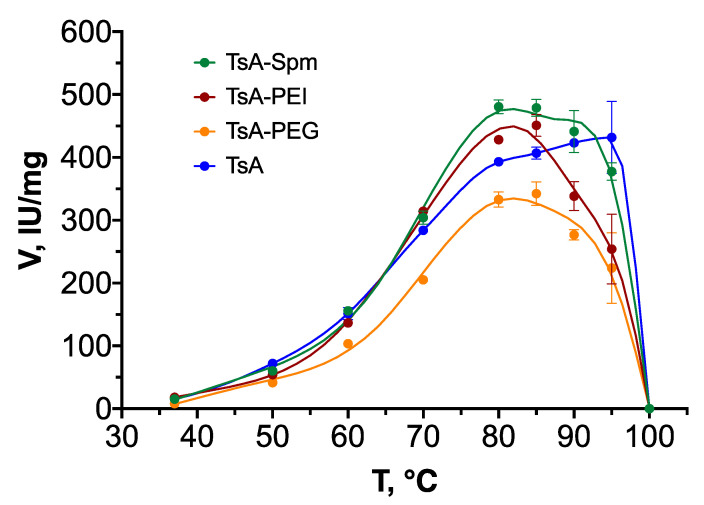
Temperature dependence of TsA and conjugate activity. C(L-Asn) = 20 mM, pH 7.5, final concentration of TsA and conjugates 0.0075 mg/mL (0.2 μM).

To examine the impact of different polymers on the activity and stability characteristics of TsA, the enzyme activity and conjugates were assessed across a temperature range of 37–100 °C at pH 7.5 (Figure 7).

The results indicate that native TsA exhibits the highest activity within the temperature range of 90–95 °C, while for conjugates, the temperature range at which maximum activity is observed decreases to 80–85 °C. At 90–95 °C, TsA has an activity of 430 IU/mL, whereas for conjugates, the activity decreases to 220–380 IU/mL. Interestingly, at 80–90 °C, TsA-Spm demonstrated the highest activity among all the preparations, 480 IU/mL.

### 2.4. Thermal Inactivation Mechanism and Aggregation of TsA Conjugates

**Figure 8:**
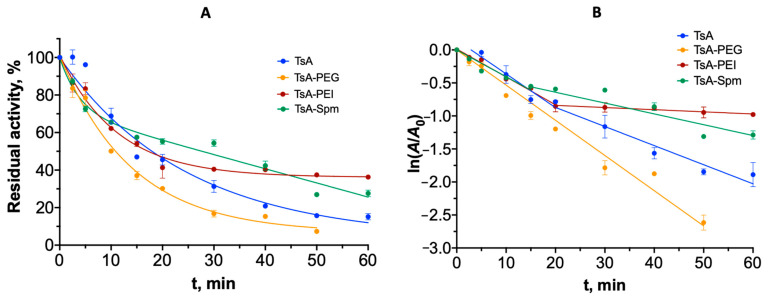
Thermal inactivation curves of TsA and conjugates at 88 °C. (**A**) Dependencies of residual enzyme activity on incubation time; (**B**) thermo-inactivation curves in semilogarithmic (first-order) coordinates.

To study the thermostability of TsA variants and the influence of the conjugation on the thermoinactivation mechanism of TsA, we determined the residual activity dependencies on the duration of incubation of the enzyme at temperatures close to the phase transition temperature (88 °C) [21]. Figure 8A shows the thermal inactivation curves for TsA and its conjugates. To study the mechanism of thermal inactivation, the resulting curves were analyzed in semilogarithmic coordinates (corresponding to first-order inactivation reaction) (Figure 8B). A deviation from first-order linearization would indicate a multimolecular mechanism of inactivation—aggregation. Equation of semi-logarithmic dependency:

$$ln\left(\frac{A}{A_{0}}\right)=-k_{in}\cdot t$$

where *A* is the enzyme activity within a given time interval; *A*_0_ is the activity at zero time; and *k*_in_ is the inactivation constant of the first-order reaction. 

Table 5 shows the resulting inactivation constants (*k*_in_). Apparently, the inactivation mechanism of TsA changes as a result of the formation of TsA conjugates with polymers of different structures. In the time interval up to 20 min, a first-order inactivation constant is observed for all of the TsA variants studied; the *k*_in_ of native TsA is 0.053 min^−1^. The conjugation of TsA to PEI or Spm has a significant stabilization effect on the enzyme (Figure 8). The *k*_in_ values decrease to 0.042 min^−1^ and 0.036 min^−1^ for TsA-PEI and TsA-Spm conjugates, respectively. At the second time interval (incubation time of 20 min) in the case of native enzyme, TsA-PEI, and TsA-Spm, the deviation from first-order linearization is observed, indicating a multimolecular mechanism of inactivation, and the enzyme aggregation appears. In the inactivation interval (20–60 min), the inactivation constant for TsA-Spm and TsA-PEI decreased by an order of magnitude to 0.003 min^−1^, compared to 0.03 min^−1^ for TsA. Notably, for TsA-Spm TsA-PEI, the quite high activity level was maintained after further incubation (from 20 min to 1 h). TsA-PEI exhibited the highest residual activity after 60 min of incubation (35% vs. 15% for TsA). For the native enzyme, visual aggregation is observed in this time interval with precipitation. 

For TsA-PEG, linearity is observed in first-order semi-logarithmic coordinates for the whole time interval studied. It appears that PEG has a protective effect on the enzyme, preventing its aggregation. However, low residual activity is observed after 60 min of incubation (15% from the initial level).

**Figure 9:**
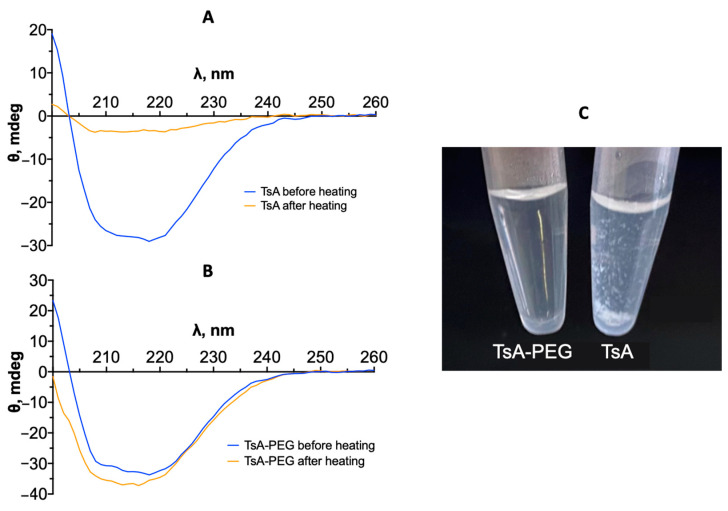
CD spectra before and after 30 min of heating at 88 °C for (**A**) native TsA and (**B**) TsA-PEG. (**C**) Photo of solutions with a concentration of 0.5 mg/mL (0.013 mM) in 10 mM PBS after 30 min of heating at 88 °C.

To analyze the distinctive features of TsA-PEG thermoinactivation in more detail, CD spectra of both the conjugate and native enzyme solutions were recorded before and after incubating the enzyme at 88 °C for 30 min (Figure 9). The quantitative spectral analysis is presented in Table 6.

The drastic decrease in the intensity of the characteristic protein peak indicated that the secondary structure of the native enzyme was generally destroyed after heating the TsA solution (Figure 9A). A precipitate was observed in the solution for both TsA-PEI and TsA-Spm conjugates, similar to that of the native enzyme. With regard to the TsA-PEG conjugate, the CD spectra remain practically unchanged (Figure 9B). This observation is further supported by the unchanged content of secondary structures, as shown in Table 6. In the case of TsA, the percentage of α-helices decreased from 33% to 9%, while the percentage of antiparallel β-sheets increased from 8% to 15%. These changes are known to be associated with multimolecular interaction and protein aggregation [21,22]. Upon heating, the TsA solution exhibited a white precipitate, which suggest protein aggregation (Figure 9C). Visual differences were observed between the TsA and TsA-PEG solutions. In contrast, the TsA-PEG solution was soluble (no precipitate was observed). However, after heating, TsA-PEG had low residual activity.

### 2.5. Changes in the Tertiary Structure of TsA and Conjugates during Thermal Denaturation

**Figure 10:**
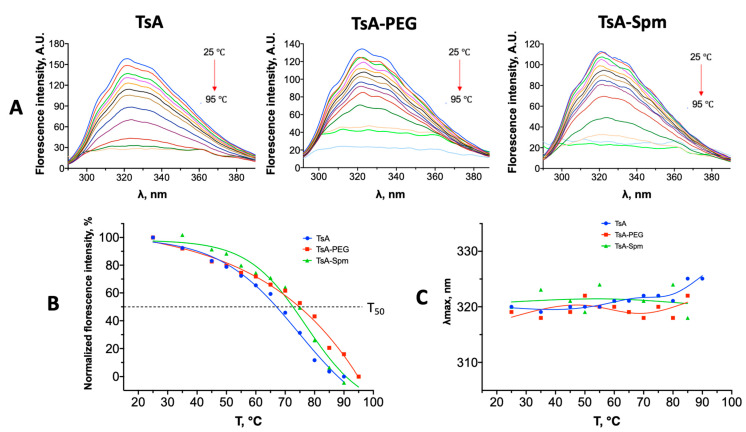
(**A**) Fluorescence spectra for L-ASNases TsA and its conjugates—TsA-PEG and TsA-Spm, heated from 25 to 95 °C; (**B**) temperature dependence of normalized fluorescence intensity at 320 nm. Protein concentration in the samples—0.013 mM; (**C**) wavelength dependencies at the maximum intensity on the heating temperature.

We found that while the secondary structure of the native enzyme changes dramatically during thermal incubation (88 °C), the secondary structure of the PEGylated enzyme mainly remained unchanged. So, the denaturation is probably accompanied by a change in the tertiary structure. Therefore, the effect of the conjugates on the thermostability and aggregation stability of the enzyme and its conjugates was studied by fluorescence spectroscopy (Figure 10). The thermograms for both the conjugates and native enzyme were obtained over a temperature range of 25 to 95 °C. The fluorescence spectra of TsA show three main peaks (Figure 10A). One corresponds to a tyrosine residue (305 nm), while the other two allow the distinction of two tryptophans in different environments, a hydrophobic one at 320 nm and a more hydrophilic one at 335 nm. From the graph in Figure 10B, the temperatures, T_50_, at which the fluorescence parameters change by 50% of the initial fluorescence intensity value (T_50_, Table 7) were determined. Changes in the fluorescence spectra were observed as a result of the unfolding at the tertiary structure level of the protein. It was found that the T_50_ for TsA was 68 °C, while for the conjugates, the decrease in intensity was less pronounced. The T_50_ for TsA-PEG and TsA-Spm was found to be 7–8 °C higher, indicating that the polymers have a stabilizing effect on the tertiary structure. At temperatures above 85 °C, there is a transfer of aromatic amino acid residues from the protein’s interior to the solution, as evidenced by the increase in emission wavelength maximum and the quenching of fluorescence.

## 3. Discussion

The modification of L-ASNases with different polymers could enhance their physicochemical and biocatalytic properties for industrial and medical applications. Our studies aimed to improve the catalytic parameters of the enzyme via modification and also to increase its stability to aggregation.

To date, the most common approach for the regulation of the catalytic parameters of the enzyme and the increase in its stability is PEGylation. To obtain a stable bond between proteins and PEG, activated PEG derivatives containing heterocyclic compounds, such as cyanurchloride and succinimide, are used, allowing a reaction between PEG and protein at mild pH and temperature values. PEGylation is a well-developed technology and is used in biopharmaceuticals to increase stability and solubility and improve the immunological properties of biologically active compounds. Among pegylated enzyme-based preparations, hydrolase-class enzymes are most widely used. Relevant examples include PEG-arginase, PEG-uricase, PEG-carboxyoxidase A, PEG-staphylokinase, PEG-glutaminase, PEG-asparaginase, etc. [23,24]. Recently, asparaginase from E. chrysanthemy has also been pegylated. Now, this drug is in the stage of preclinical studies. PEG is known to assist in stabilizing the enzyme’s secondary structure and preserving its natural conformation. The polymer is used for the modification of L-ASNase, and PEGylated L-ASNase preparations are already commercially available [25,26,27]. 

Additionally, polycations are commonly used to modify enzymes, the effects of which vary depending on the polymer’s structure and the enzyme itself [28,29]. Polyelectrolytes can be used to change the charge of an enzyme, which can affect its properties. For instance, by modifying the charge near the active center, enzyme activity can be altered. This approach can be advantageous for achieving higher activity within the desired pH range. For example, polyamines have been used to shift the pH optimum of an enzyme toward more neutral values to increase the cytotoxicity of RrA [17]. It has been suggested in the literature that the cytotoxicity of enzyme preparations can be influenced by increasing L-Asn hydrolysis activity at pH values at which tumor cells grow. Furthermore, the adsorption of the enzyme on tumor cell membranes can be further affected by polycations, which can have an impact on cytotoxicity.

Recently, polyamines, such as chitosan and PEI derivatives, have been found to be effective at modifying RrA, possibly contributing to the improvement in the abovementioned properties [17]. However, since enzymes from different sources have different structures and properties, the effects of polymers may also differ, as in the case of EwA and RrA [18]. This study aimed to compare the effects of charged (Spm and PEI) and uncharged (PEG) polymers on hyperthermophilic TsA with the effects on other L-ASNases that we have recently studied.

Upon studying the influence of the conjugate formation with polymers, an important parameter affecting the catalytic properties of the enzyme is the oligomeric composition. It is believed that L-asparaginases from hyperthermophilic sources can exist as monomeric or dimeric forms [30,31,32]. Here, the chromatograms for the TsA enzyme studied reveal only a monomeric form; the dimer is not formed under the conditions studied. SDS-PAGE analysis of the TsA structure revealed a major band at approximately 37 kDa, which corresponds to the monomeric form (Figure S1). Additionally, a very minor band at around 80–90 kDa was observed, which is likely to be the dimeric form. This form is more manifested when the enzyme is incubated with oxidized glutathione (Figure S1A). Native electrophoresis (Figure S1C) also shows a major band corresponding to the monomeric form of the enzyme and a minor band with a higher molecular weight, corresponding to the dimer. The monomeric form still predominates, with a content of at least 90%. To initiate dimeric form formation and the aggregation process, the enzyme was incubated at 90 °C up to 6 h, and as a result, the bands corresponding to the dimeric form and aggregates were detected using SDS-PAGE (Figure S1B). However, it is worth noting that the dimeric form is not observed in the HPLC chromatogram (Figure 2). To further examine the oligomeric composition, TIC and UV chromatograms (Figure S2) as well as mass spectra of TsA solutions (Figure S3) were obtained. Based on the TIC and UV chromatograms and the analysis of the mass spectra (Figure S3), it appears that only the monomeric form is present in solution.

Analysis of TsA conjugates by HPLC chromatography as well as SDS-PAGE analysis were carried out to control the oligomeric composition of the TsA upon conjugate formation. For TsA-PEG in the HPLC chromatogram (Figure 2), the main major peak was assigned to the PEGylated enzyme. SDS-PAGE analysis for TsA-PEG also showed one major blurred band around 73–94 kDa (Figure S4). This band is most likely attributed to the monomer with PEG chains, which consists of approximately 5–7 PEG chains (25–35 kDa) and TsA monomers (37.5 kDa), resulting in a total of 62–72 kDa. 

It is also important to mention that conjugates of L-asparaginase with polycations typically do not have significant differences in secondary structure from the native enzyme [17]. In the case of TsA, there was no significant change in the CD spectra after conjugate formation. Therefore, it can be concluded that neither PEGylation nor modification with polyamines affects the secondary structure of TsA. 

When analyzing the thermal inactivation for native TsA and conjugates with PEI and Spm, we observed initial regions within 20 min corresponding to first-order inactivation. After 20 min of thermoinactivation, the increase in the order of the inactivation constant is observed. This suggests a multimolecular process—aggregation—which was also observed by other methods such as CD, fluorescence, and visual observation. So, the enzymes inactivated via denaturation, followed by aggregation processes. All samples studied, except TsA-PEG, exhibit aggregation after a 20 min interval.

The fluorescence data appear to correlate with the thermoinactivation data (in terms of catalytic activity) for the samples studied. The thermograms show that, in the initial phase, the loss of the tertiary structure of the enzymes is predominant, followed by the destruction of the secondary structure (at temperatures above 80–85 °C according to CD spectroscopy) and the formation of aggregates, some of which are insoluble (Figure 9C). In the case of TsA-PEG, no precipitation was observed. However, a loss of activity was detected, which seems to be mainly linked to the disruption of the tertiary structure.

As a result, the impact of polyelectrolytes on various L-ASNases with different structures and properties may vary. For example, when studying the thermoinactivation of the polyelectrolyte complex of EwA with PEI, it was found that the thermoinactivation constant for this PEC is higher than that for the native enzyme [18]. The destabilization of the EwA structure can be attributed to the excessive positive charge of PEI. On the other hand, RrA complexes with PEI exhibited higher thermostability, as indicated by a decrease in the thermal inactivation constant. It has been previously observed that polycations may also contribute to the stabilization of the quaternary structure of RrA [17]. A decrease in the aggregation degree and *k*_in_ of the TsA-PEI conjugate was also observed in comparison with those of TsA. Therefore, conjugation with Spm and PEI significantly stabilizes the enzyme against thermoinactivation. The thermoinactivation of the TsA-PEG conjugate, which alters the inactivation mechanism, is an interesting result. For this conjugate, no protein aggregation was observed after heating at 88 °C. However, in the case of TsA-PEG *k*_in_, there was a decrease in activity, although the conjugate retained its secondary structure but can lose its tertiary structure. This phenomenon has also been observed for RrA [21]. In the case of TsA, PEG may help protein particles repel each other better at elevated temperatures due to the presence of long hydrophilic PEG chains.

Therefore, our study demonstrated the prospects of the approach for regulating the biocatalytic properties and stability of enzymes of the biotechnological application on the example of the L-asparaginase TsA. The developed approach is based on the formation of conjugates of enzymes with PEG and grafted copolymers of a branched structure based on polycations. The polyelectrolyte nature of the polymers promotes their multipoint electrostatic interaction with the protein surface, which makes it possible to modulate the catalytic properties of the enzyme, including by shifting the pH of the optimum enzyme activity to the range of physiological pH values (pH 7.5–8.5). Optimization of the molecular architecture and composition of conjugates led to an increase in the catalytic efficiency of the enzyme (Vmax/KM) up to 1.5 times (Table 4). The most striking effect on the catalytic activity of the enzyme is the modification with spermine. One of the main reasons for the change in conjugate activity in comparison with the native enzyme is the shift in the optimum pH of enzyme activity toward physiological pH values. In addition, the formation of conjugates with PEG leads to an increase in thermal stability and stability to thermoinactivation, compared with the native enzyme, reducing its inactivation constant by ~1.5 times, depending on the composition of the conjugate.

## 4. Materials and Methods

### 4.1. Enzyme Preparation and Chemicals

The enzyme preparation of LASNase from the microorganism *Thermococcus sibiricus* was performed according to a method described previously [8]. The L-ASNase gene of *Thermococcus sibiricus* (sequence 1510265–1511260 https://www.ncbi.nlm.nih.gov/nuccore/NC_012883.1, accessed on 4 April 2024, protein GenBank accession No. WP_015849943.1) was synthesized by TWIST Bioscience (Twist Bioscience HQ, San Francisco, CA, USA). The synthesized gene was cloned and inserted into the pET-28a(+) vector under the control of the T7 promoter. The constructed vector was subsequently transformed and expressed in *E. coli* BL21 (DE3) cells.

Selected recombinant *E. coli* clones were grown as previously described [17]. To culture the cells harboring the plasmids, 0.05 mg/mL of kanamycin was added to the medium. Expression of the target protein was induced by lactose added to the expressed culture at an OD600 of 1.9 to a final concentration of 0.2%. The cells were grown for another 17–20 h and then centrifuged at 4000× *g* for 15 min.

All the enzyme purification steps were carried out at +4 °C. Five grams of biomass was suspended in 50 mL of buffer (20 mM sodium phosphate buffer (pH 7.2), 1 mM glycine, and 1 mM EDTA) and ultrasonicated. Cell debris and nondegraded cells were removed by centrifugation (35,000× *g*, 30 min). The supernatant containing the target enzyme was applied to SP-Sepharose columns. The proteins were eluted with a linear gradient of 0–1.0 M NaCl. The protein content of the fractions was checked by absorbance at 280 nm and by measuring enzyme activity. Ultrafiltration, desalting, and buffer exchange were performed using Amicon membranes (Millipore, Burlington, MA, USA). The samples were frozen and stored at −20 °C.

The following reagents were used in this work: L-asparagine (BioChemica, Billingham, UK), phosphate-salt buffer and sodium tetraborate (Na_2_B_4_O_7_-10H_2_O, (PBS; Eco-Service, Moscow, Russia), citric acid and NaOH (Reachim, Moscow, Russia), DMSO (Sigma-Aldrich, Burlington, NJ, USA).

### 4.2. Synthesis and Purification of L-Asparaginase Conjugates

The following reagents were used for synthesis: linear polyethyleneimine, 2 kDa (PEI; Sigma-Aldrich, St. Louis, MI, USA); spermine (Spm; Sigma-Aldrich, USA); and linear activated Methoxy PEG Succinimidyl Carboxymethyl Ester 5 kDa (PEG; JenKem, Zhengzhou, China).

L-ASNase reaction with activated PEG: To a mixture of 5 mg/mL of TsA in 10 mM PBS (pH 7.3; ECO-Service, Russia), a 15–25-fold excess of PEG was added, and the mixture was dissolved in a minimal volume of DMSO. The resulting concentration of DMSO in solution with protein did not exceed 5–10%. The resulting mixture was incubated under stirring at room temperature for 2 h. The product was purified by diafiltration on Amicon centrifuge filters^®^ (Merck-Millipore, Kenilworth, NJ, USA) with pores allowing molecules less than 30 kDa in mass to pass through. Filtration was carried out approximately 7 times. The determination of the number of PEG chains bound to the protein was carried out using IR spectroscopy. Furthermore, similar results were obtained through the application of the titration method with TNBS. Additionally, an AFM microscope (NTEGRA II, NT-MDT Spectrum Instruments, Moscow, Russia) was used to control the modification and visualize polymeric conjugates of the enzyme and compare it with non-modified form. 

L-ASNase was reacted with Woodward K reagent (WRK, Sigma-Aldrich, Burlington, NJ, USA) and polyamines. To a solution of TsA in 50 mM MES (pH 5.5), a solution of WRK reagent in HCl (Component-Reaktiv, Moscow, Russia) (pH 2.0) was added to achieve final concentrations of 1 mg/mL (0.027 mM) and 0.5 mM, respectively. The pH 5.5 of the reaction solution was controlled by MES buffer (pH 5.5). The resulting mixture was incubated for 2 h at room temperature, after which the excess WRK was removed through diafiltration. The completeness of the purification was monitored by observing the decrease in peak intensity at 245 nm. A 30-fold excess of PEI relative to the mol of enzyme was added to the purified TsA-WRK adduct in 10 mM PBS, pH 7.3. The final concentrations of ASNase and PEI in solution were approximately 1–1.5 and 1.5 mg/mL, respectively. The mixture was incubated for approximately 15–20 h at room temperature. The conjugate was subsequently purified by diafiltration approximately 4–5 times. The purity of the preparation was assessed using HPLC gel filtration on a Superdex 200 Increase 10/300 GL column in a Knauer chromatography system (Knauer, Berlin, Germany). The eluent consisted of 50 mM Tris buffer and 200 mM NaCl at pH 7.5, with an elution rate of 0.75 mL/min at 25 °C. Bovine serum albumin (BSA, Sigma-Aldrich, Burlington, NJ, USA) with a molecular mass of 66.4 kDa was used as a reference protein. The resulting conjugates were either lyophilized or frozen and stored at −20 °C.

### 4.3. L-Asparaginase Catalytic Activity Measurement

The enzymatic activity of the L-ASNases was measured on a Jasco J-815 circular dichroism (CD) spectrometer (Jasco, Tokyo, Japan) according to the method described previously [17]. The reaction was carried out by mixing solutions of L-asparagine and L-ASNase in 25 mM borate buffer at pH 9.3 to final concentrations of 1–40 mM and 0.007–0.01 mg/mL (0.2–0.3 μM), respectively. The change in ellipticity was recorded at a wavelength of 210 nm in three replicates. The reaction was performed in a 300 μL quartz cuvette with an optical path length of 1 mm in a temperature-controlled cell at 85 °C. The dependence of the initial reaction rate on the substrate concentration was plotted using Prism 8 software (GraphPad Software, San Diego, CA, USA). The values of Vmax and K_M_ were determined by linearizing the experimental data in a Lineweaver–Burk plot (or double-reciprocal plot, 1/V, 1/S_0_).

To determine the pH dependence of the native enzyme and its conjugates, a series of solutions containing 20 mM L-asparagine in 5 mM citrate–phosphate–borate buffer were prepared. The pH of the L-asparagine solutions was adjusted to a range of 5.5–11 using NaOH solution. Next, 10 μL of enzyme at a concentration of 1 mg/mL was added to 300 μL of substrate solution in the cuvette, and the activity was recorded at 37 °C using a CD spectrometer.

The temperature dependence of L-ASNase activity was determined using a Peltier cell line, PMH-428s/15, which heats CD spectrometer cells. In a typical experiment, 300 µL of a preheated substrate solution (20 mM L-asparagine, 25 mM borate buffer, pH 9.3) was used. Next, 10 µL of enzyme solution was added, and the activity was measured at temperatures ranging from 35 to 95 °C. Product accumulation curves were recorded for 100–200 s.

Thermal inactivation curves of L-ASNases were obtained using a previously described method [18]. In a typical experiment, a sample enzyme solution (0.5 mg/mL) was incubated in 10 mM PBS (pH 7.3) at 88 °C. After every 2.5–10 min of incubation, aliquots were taken and cooled for 4–5 min to room temperature. The enzymatic activity of the samples was measured at 85 °C. The obtained data were linearized in first-order reaction coordinates.

### 4.4. Registration of CD Spectra

CD spectra of the L-asparaginase solutions and purified conjugate solutions were recorded using a J-815 CD spectrometer (Jasco, Tokyo, Japan) equipped with a thermostatically controlled cell. Measurements were performed in the wavelength range of 200–260 nm at 37 °C in a quartz cuvette (l = 1 mm). Spectra were obtained by 3-fold scanning in 1 nm steps. Then, 300 μL of enzyme and conjugate samples in 10 mM PBS (pH 7.5) were added to the cuvette. The final concentration of native enzyme in the system was 0.25–1.5 mg/mL. The concentration of protein in the conjugates was determined from the graduation curve for the native enzyme. The data were analyzed using Prism 8 software (GraphPad Software, San Diego, CA, USA). Deconvolution of the spectra to analyze the content of secondary structures was performed using CDNN 2.1 software (Applied Photophysics Ltd., Surrey, UK).

### 4.5. Registration of IR Spectra

A Tensor 27 IR Fourier spectrometer (Bruker, Ettlingen, Germany) with an MCT detector was used to obtain the FTIR spectra of TsA and its conjugates. The measurements were carried out with a BioATR II thermostated cell using a single-reflection ZnSe element at 22 °C and FTIR microscope MICRAN-3. An aliquot (40 µL) of the corresponding enzyme solution (1.0–1.5 mg/mL in 10 mM sodium phosphate buffer) was applied to the internal reflection element, and the spectrum was recorded three times in the range from 3000 to 950 cm^−1^ with a resolution of 1 cm^−1^; we performed 50-fold scanning and averaging. The background was registered in the same way and automatically subtracted by the program. The resulting spectra were smoothed by the Savitzky–Golay method to a spectral resolution of 2 cm^−1^ [33]. The spectra were analyzed using Opus 7.0 software (Bruker, Ettlingen, Germany).

### 4.6. Registration of Fluorescence Spectra

Fluorescence spectra in the 290–390 nm range were obtained on a Cary Eclipse Fluorescence spectrometer (Agilent Technologies, Santa Clara, CA, USA) with an excitation wavelength of 280 nm (for Trp and Tyr excitation). TsA and conjugate solutions were prepared in 10 mM PBS to a final concentration of 1 mg/mL. The obtained solutions were heated to the required temperatures in a thermostatically controlled cell for 1–2 min. Then, measurements were performed. To determine the T_50_ parameter, graphs of the dependence of fluorescence intensity at 320 nm on temperature were plotted and normalized using Prism 8 software.

## 5. Conclusions

This study aimed to investigate the effects of charged and uncharged polymers on the chemical and physical properties of L-ASNase from the hyperthermophilic archaeon *Thermococcus sibiricus*. This L-ASNase is quite different in structure and properties from the bacterial L-ASNases *Erwinia carotovora* and *Rhodospirillum rubrum* that we previously studied. The results showed that the effects of PEG, PEI, and Spm on TsA were similar to their effects on RrA. PEG did not significantly affect the catalytic parameters under different conditions or the rate of thermoinactivation. However, it prevented the aggregation of the enzyme during thermodenaturation. This property could be useful in the food processing industry when using L-ASNase for the processing of starch-containing foods. Additionally, PEI and Spm were found to increase enzyme activity at lower pH ranges, which could be relevant for enhancing the cytotoxicity of drugs. The addition of polycations to the enzyme preparation reduces the rate of thermoinactivation of TsA and can prevent protein aggregation. The results obtained open the prospects to the industrial application of L-asparaginase enzyme preparations.

## Figures and Tables

**Table 1:** Characterization of polymers used for conjugation with TsA.

Polymer	MM, Da	Origins	Structure	ζ-Potential, mV (at pH 7.0)	Ref.
Polyethylenimine	2000	Synthetic	Linear	20	[19]
Spermine	202	Natural	Linear	-	
Polyethyleneglycol	5000	Synthetic	Linear	−(4–6)	[20]

**Table 2:** Retention times for the main fractions of lysozyme, BSA, native TsA, and conjugate TsA-Spm and TsA-PEG.

Samples	t_R_, min	MM, kDa
Lysozyme	27.6	14.3
BSA	18.2	66.4
TsA	20.2	37.5
TsA-Spm	19.5	40.4
TsA-PEG	13.9	>90

**Table 3:** Percentage content of native enzyme secondary structure elements and TsA-PEG, TsA-PEI, and TsA-Spm conjugates, calculated via CDNN program.

Secondary Structures	TsA	TsA-PEG	TsA-PEI	TsA-Spm
Helix	33.4 ± 1.6	32.2 ± 1.8	33.6 ± 2.1	34.3 ± 0.5
Antiparallel	8.2 ± 0.7	8.3 ± 0.2	8.2 ± 0.8	8.0 ± 0.3
Parallel	8.8 ± 0.4	9.3 ± 0.8	8.8 ± 0.6	8.7 ± 0.2
Beta-Turn	16.8 ± 0.3	16.9 ± 0.2	16.7 ± 0.3	16.6 ± 0.1
Rndm. Coil	32.8 ± 0.7	34.2 ± 1.6	33.0 ± 1.3	32.6 ± 0.3

**Table 4:** K_M_ and Vmax values for native TsA and conjugates; 25 mM borate buffer, pH 8.5, 85 °C.

	K_M_, mM	Vmax, IU/mg	V_max_/K_M_
TsA	6.1 ± 1.3	470 ± 40	77
TsA-PEG	6.3 ± 0.8	460 ± 20	73
TsA-PEI	4.1 ± 0.7	520 ± 20	126
TsA-Spm	6.2 ± 1.6	710 ± 30	115

**Table 5:** Values of TsA and conjugate inactivation.

	*k*_in_, min^−1^ (0–20 min)	*k*_in_, min^−1^ (20–60 min)
TsA	0.052 ± 0.002	0.029 ± 0.002
TsA-PEG	0.053 ± 0.002	0.053 ± 0.002
TsA-PEI	0.042 ± 0.003	0.003 ± 0.001
TsA-Spm	0.036 ± 0.001 *	0.019 ± 0.001 *

* The constants for 0–15 and 15–60 min are indicated.

**Table 6:** Percentage content of secondary structure elements of TsA and TsA-PEG, calculated via CDNN program. * The data were calculated for the protein that remains in solution, which constitutes approximately 10%.

Secondary Structures	TsA		TsA-PEG	
	Before Heating	After * Heating	Before Heating	After Heating
Helix	33.4 ± 1.6	8.8 ± 1.2	32.2 ± 1.8	32.4 ± 1.6
Antiparallel	8.2 ± 0.7	15.2 ± 0.3	8.3 ± 0.2	8.6 ± 0.8
Parallel	8.8 ± 0.4	15.7 ± 0.5	9.3 ± 0.8	9.0 ± 0.3
Beta-Turn	16.8 ± 0.3	17.4 ± 0.4	16.9 ± 0.2	17.1 ± 0.4
Rndm. Coil	32.8 ± 0.7	43.2 ± 1.1	34.2 ± 1.5	33.1 ± 0.3

**Table 7:** The temperatures at which no more than 50% of the initial fluorescence intensity remains for TsA, TsA-PEG, and TsA-Spm.

Sample	T_50_, °C
TsA	68
TsA-PEG	76
TsA-Spm	75

## Data Availability

The data presented in this study are available upon request from the corresponding author.

## Supplementary Materials

The following supporting information can be downloaded at https://www.mdpi.com/article/10.3390/ijms25084174/s1.
