# Supplementary material for: L-Asparaginase Conjugates from the Hyperthermophilic Archaea Thermococcus sibiricus with Improved Biocatalytic Properties
Source: Int J Mol Sci. 2024 Apr 10;25(8):4174. doi: 10.3390/ijms25084174 (PMC11050321; doi:10.3390/ijms25084174)
Supplement: Supplementary file 1 [file ijms-25-04174-s001.zip › ijms-2877379-supplementary.pdf]

Supplementary Materials

L-asparaginase conjugates from the hyperthermophilic archaea *Thermococcus sibiricus* with improved biocatalytic properties

Natalia V. Dobryakova [1,2], Maria V. Dumina [1], Alexander A. Zhgun [1], Marina V. Pokrovskaya [3], Svetlana S. Aleksandrova [3], Dmitry D. Zhdanov [1,3] and Elena V. Kudryashova [1,2,*]

[1] Group of Fungal Genetic Engineering, Federal Research Center "Fundamentals of Biotechnology of the Russian Academy of Sciences", 117312 Moscow, Russia; natdobryak@gmail.com (N.V.D.); duminamaria@gmail.com (M.V.D.); zzhgun@mail.ru (A.A.Z.)
[2] Chemical Faculty, Lomonosov Moscow State University, Leninskie Gory St. 1, 119991 Moscow, Russia
[3] Laboratory of Medical Biotechnology, Institute of Biomedical Chemistry, 119121 Moscow, Russia, ivan1190@yandex.ru (M.V.P.); v-aleksandrov@yandex.ru (S.S.A.), zhdanovdd@gmail.com (D.D.Z.);
* Correspondence: helenakoudriachova@yandex.ru

Content

1. Figure S1. SDS-PAGE and native electrophoresis for the TsA enzyme.

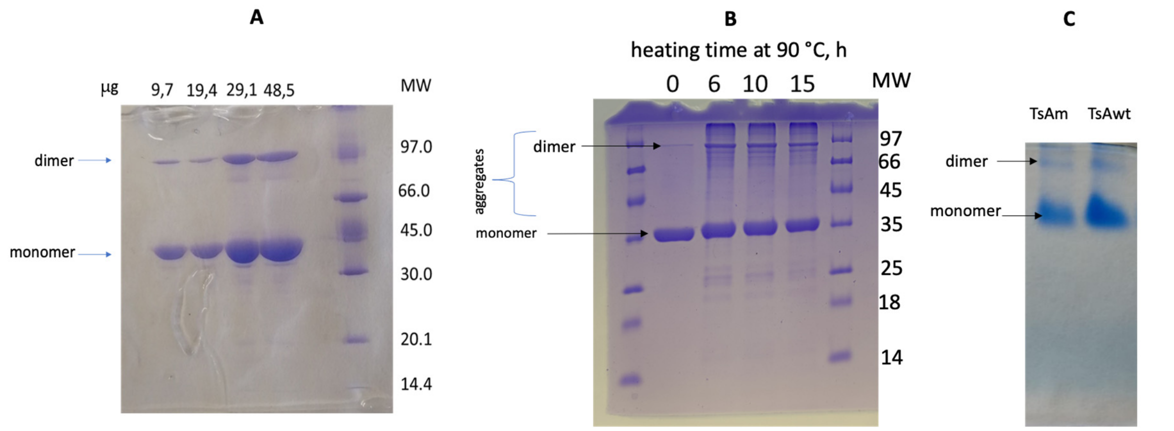

Figure S1. SDS-PAGE for the TsA enzyme with oxidised glutathione (A) and heating times at 90 °C (B). Native electrophoresis of wild-type TsA and the mutant (C).

2. Figure S2. TIC and UV chromatograms of TsA

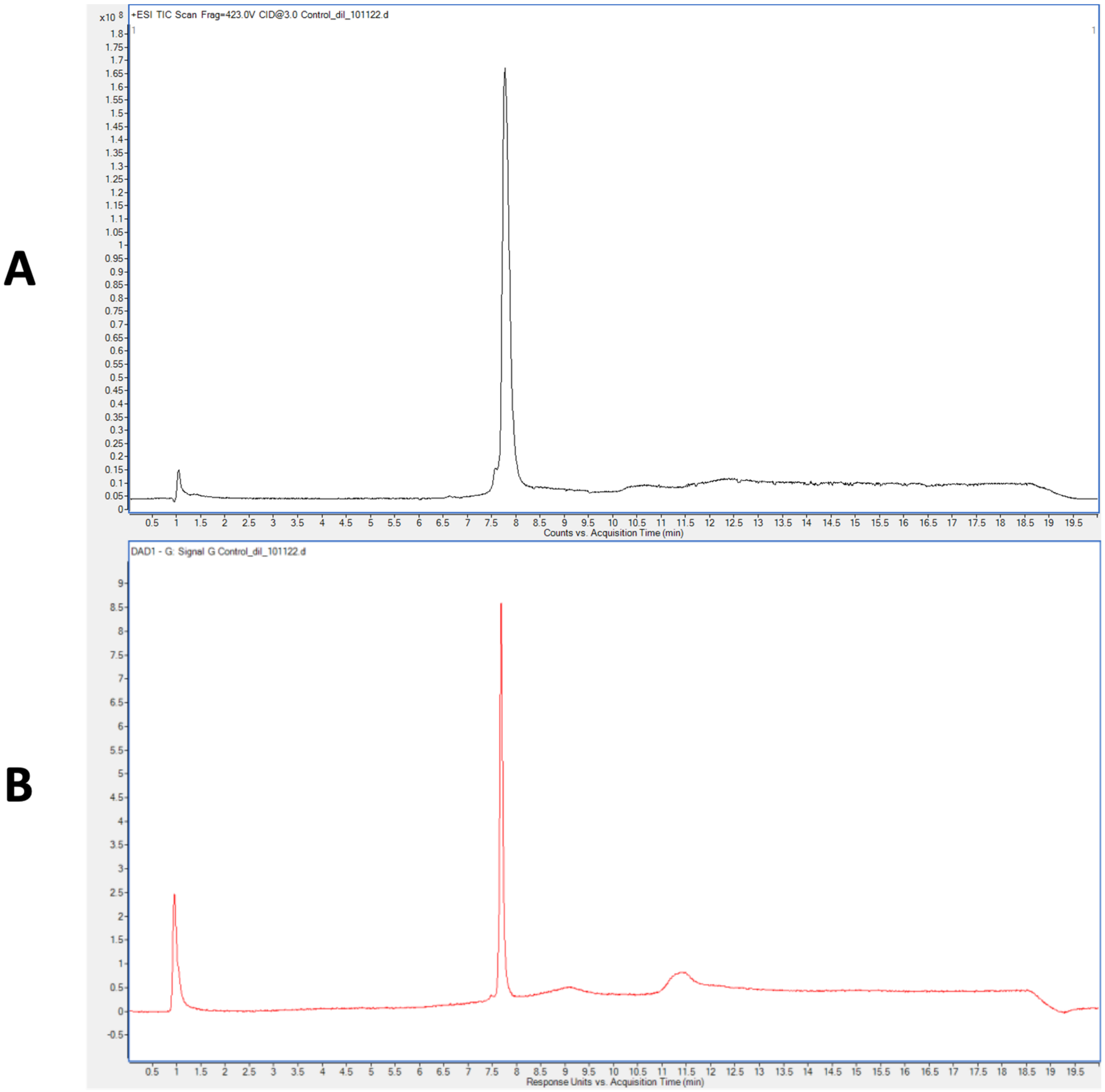

Figure S2. *(A)* Results of total ion current chromatogram (TIC chromatogram), 0.53 μg TsA; (B) UV chromatogram, 0.53 μg TsA, 280 nm.

3. Figure S3. Mass spectra of the TsA.

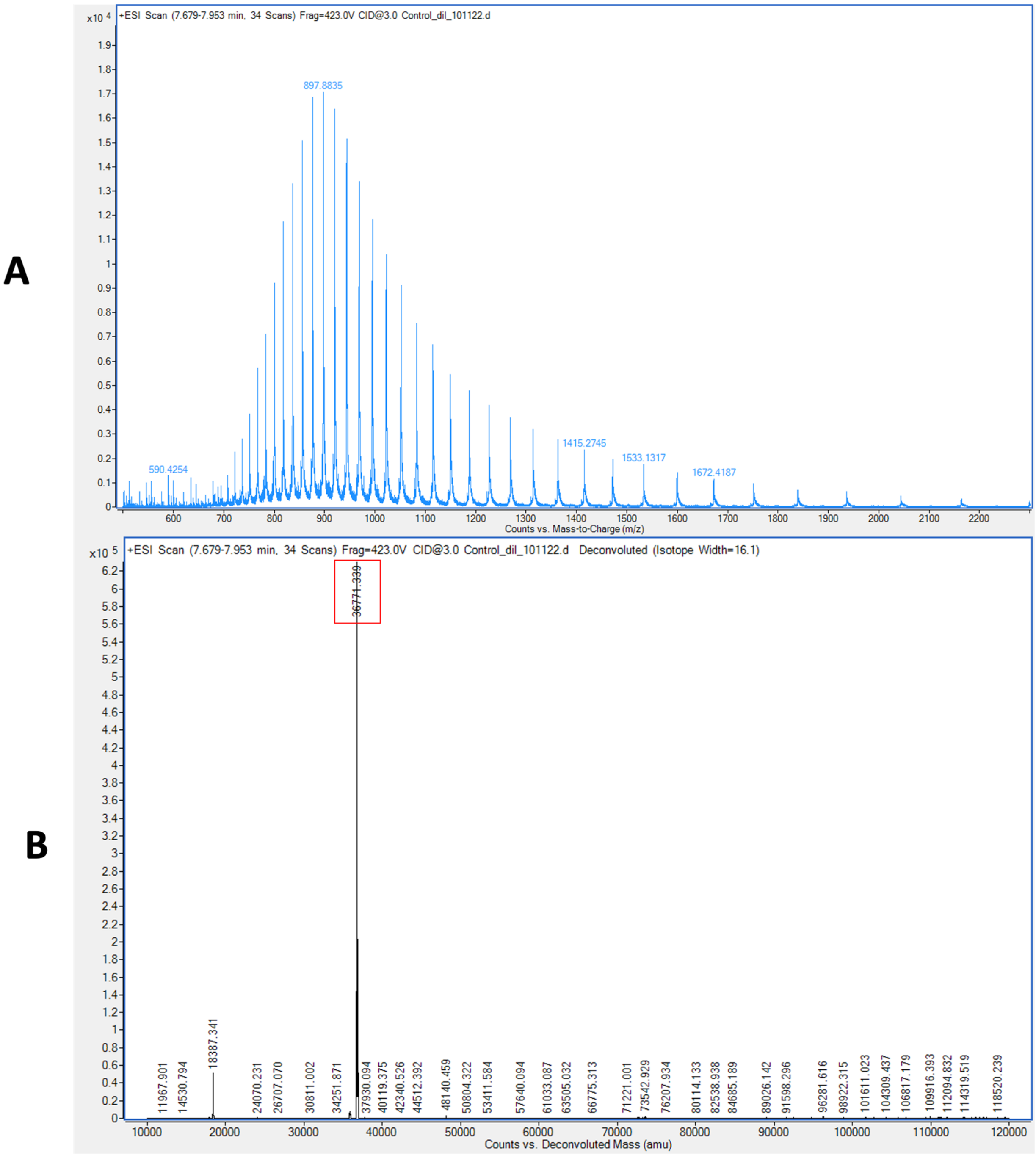

Figure S3. (A) Extracted mass spectrum of 0.53 μg of TsA over a chromatography interval of 7.6 to 7.9 minutes; (B) Deconvolution of the extracted mass spectrum of 0.53 μg of TsA over a chromatography interval of 7.6 to 7.9 minutes.

4. Figure S4. SDS-PAGE for TsA and TsA-PEG.

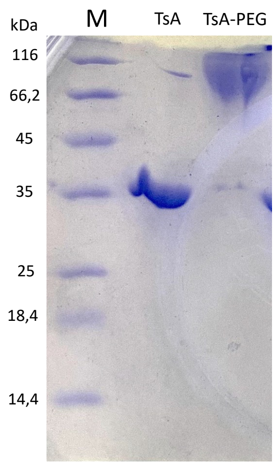

Figure S4. SDS-PAGE for TsA and TsA-PEG.
